# Integration of metabolomics and machine learning revealed tryptophan metabolites are sensitive biomarkers of pemetrexed efficacy in non‐small cell lung cancer

**DOI:** 10.1002/cam4.6446

**Published:** 2023-08-21

**Authors:** Runbin Sun, Fei Fei, Min Wang, Junyi Jiang, Guangyu Yang, Na Yang, Dandan Jin, Zhi Xu, Bei Cao, Juan Li

**Affiliations:** ^1^ Phase I Clinical Trials Unit Nanjing Drum Tower Hospital, Affiliated Hospital of Medical School, Nanjing University Nanjing China; ^2^ Department of Pharmacy Nanjing Drum Tower Hospital, Affiliated Hospital of Medical School, Nanjing University Nanjing China; ^3^ General Medical Department Nanjing Drum Tower Hospital, Affiliated Hospital of Medical School, Nanjing University Nanjing China

**Keywords:** drug resistance, machine learning, NSCLC, pemetrexed, pharmacometabolomics

## Abstract

**Background:**

Anti‐folate drug pemetrexed is a vital chemotherapy medication for non‐small cell lung cancer (NSCLC). Its response varies widely and often develops resistance to the treatment. Therefore, it is urgent to identify biomarkers and establish models for drug efficacy evaluation and prediction for rational drug use.

**Methods:**

A total of 360 subjects were screened and 323 subjects were recruited. Using metabolomics in combination with machine learning methods, we are trying to select potential biomarkers to diagnose NSCLC and evaluate the efficacy of pemetrexed in treating NSCLC. Furtherly, we measured the concentration of eight metabolites in the tryptophan metabolism pathway in the validation set containing 201 subjects using a targeted metabolomics method with UPLC‐MS/MS.

**Results:**

In the discovery set containing 122 subjects, the metabolic profile of healthy controls (H), newly diagnosed NSCLC patients (ND), patients who responded well to pemetrexed treatment (S) and pemetrexed‐resistant patients (R) differed significantly on the PLS‐DA scores plot. Pathway analysis showed that glycine, serine and threonine metabolism occurred in every two group comparisons. TCA cycle, pyruvate metabolism and glycerolipid metabolism are the most significantly changed pathways between ND and H group, pyruvate metabolism was the most altered pathway between S and ND group, and tryptophan metabolism was the most changed pathway between S and R group. We found Random forest method had the maximum area under the curve (AUC) and can be easily interpreted. The AUC is 0.981 for diagnosing patients with NSCLC and 0.954 for evaluating pemetrexed efficiency.

**Conclusion:**

We compared eight mathematical models to evaluate pemetrexed efficiency for treating NSCLC. The Random forest model established with metabolic markers tryptophan, kynurenine and xanthurenic acidcan accurately diagnose NSCLC and evaluate the response of pemetrexed.

## 1 INTRODUCTION

The incidence rate of lung cancer is second only to breast cancer and is the leading cause of cancer‐related deaths worldwide.^1^ Non‐small cell lung cancer (NSCLC) represents approximately 80% of all lung cancers. Previously, surgery, chemotherapy, and radiotherapy were recommended for NSCLC patients.^2^ However, most advanced NSCLCs become resistant to current treatments and eventually progress to end‐stage lung cancer.^3^ Pemetrexed is a first‐line treatment for locally advanced and metastatic NSCLC in patients with nonsquamous histology combined with cisplatin or carboplatin. It is a chemotherapy drug chemically similar to folic acid and can function as folate antimetabolite. By inhibiting the formation of purine and pyrimidine nucleotides, pemetrexed prevents the formation of DNA and RNA, thus inhibiting the growth and survival of cancer cells.^4^ However, over 90% of NSCLC patients who received chemotherapy will develop spontaneous or acquired resistance that hinders clinical efficacy after long‐term use. The molecular mechanisms of pemetrexed tolerance include higher expression of pemetrexed targeting enzymes, enhanced DNA repair systems, cancer stemness, impaired drug uptake, and reinforced drug elimination.^5^, ^6^ Improving the efficiency of pemetrexed and reducing its resistance is a valuable method for appropriate drug administration to NSCLC patients.

Pharmacogenomics focuses on how genomic variations among patients affect the efficacy or safety of drug treatment.^7^ It was found that KRAS mutation can predict the sensitivity to pemetrexed‐based chemotherapy.^8^ The expression of thymidylate synthase,^9^ folic acid phenotype,^10^ glucocorticoid receptor,^11^ ABCC11 transporter^12^ and βIII tubulin expression^13^ are considered determinants of pemetrexed sensitivity. High expression of miR‐22^14^ and miR‐25^15^ are correlated with the lack of response in pemetrexed treated NSCLC patients. The accuracy of these methods requires further improvement. Developing new pretreatment pemetrexed resistance prediction and efficiency evaluation methods is extremely important.

Metabolomics technology is widely used to diagnose diseases, discover drug treatment targets, and evaluate drug safety.^16^, ^17^, ^18^, ^19^, ^20^, ^21^ Pharmacometabolomics is based on the differences and characteristics of the material basis of individual organisms to predict the disposal, exposure levels and effects of drugs in the body, providing new ideas for personalized medicine.^22^ Metabolic reprogramming is one of the essential characteristics of tumors and is closely related to tumor drug resistance. Glucose metabolism, glutamine metabolism, and lipid metabolism are the most significantly changed metabolic pathways in tumor cells.^23^ As a folic acid antimetabolite, pemetrexed interferes with synthesizing thymine and purine nucleotides. The basal metabolic capacity of cells is very likely to affect the therapeutic effect of pemetrexed. Evaluation of disease staging and chemotherapeutic response in NSCLC from patient tumor‐derived metabolomic data is of significant meaning. A predictive model based on nuclear magnetic resonance‐based metabolomics was established to indicate lung cancer presence and patient survival.^24^ In another study, a set of six metabolites (hypoxanthine, inosine, L‐tryptophan, indoleacetic acid, acyl‐carnitine C10:1, and lysoPC[18:2]) were chosen as potential biomarkers for NSCLC, and the area under the curve (AUC) value was 0.99.^25^ Diacetylspermine is a novel prediagnostic serum biomarker for NSCLC.^26^ However, these studies only focus on diagnosing NSCLC, and the biomarker of pemetrexed efficacy is mainly unknown. Further research is needed to discover new sensitive markers for evaluating the effectiveness of pemetrexed and establish models with high accuracy to increase the efficiency of pemetrexed and promote precision treatment for NSCLC.

Machine learning algorithms include unsupervised and supervised learning, mainly aimed at classification, and regression. With the advent of big data, artificial intelligence, and machine learning algorithms have become increasingly widely used in medicine and health, such as disease diagnosis, personalized treatment, drug design, clinical trial research, and epidemic outbreak prediction. Metabolomics technology can reveal the metabolic characteristics of lung cancer.^27^ In the clinical diagnosis and treatment process of primary lung cancer, cancer‐related serum markers have been used as a recommended inspection item for outpatient lung cancer patients.^28^ In this project, we will further analyze the metabolic characteristics of drug resistance in NSCLC and combine machine learning with serum metabolomics data obtained from pemetrexed sensitive/resistant NSCLC patients to build an NSCLC pemetrexed efficiency evaluation model with high sensitivity and specificity.

This study aims to integrate pharmacometabolomics and machine learning methods to reveal the metabolic characteristics of NSCLC, screen biomarkers of pemetrexed efficiency, validate them using targeted metabolomics and establish a mathematical model to evaluate pemetrexed response, and further try to clarify the metabolic mechanism of pemetrexed resistance and provide reference and basis for precise and personalized treatment of NSCLC.

## 2 MATERIALS AND METHODS

### 2.1 Chemicals

Methoxyamine, N‐methyl‐N‐trimethylsilyl‐trifluoro‐acetamide (MSTFA) and 1% trimethylchlorosilane (TMCS) were purchased from Sigma‐Aldrich (Shanghai, China). Methanol, acetonitrile, and n‐Heptane were purchased from Merck KGaA (Darmstadt, Germany). Stable‐isotope‐labeled 5‐^13^C‐glutamine and myristic‐1,2‐^13^C_2_ acid were purchased from Cambridge Isotope Laboratories (Andover, MA, USA). Tryptophan, kynurenine, xanthurenic acid, tryptamine, indolelactic acid, melatonin, quinolinic acid, 3‐indolepropionic acid and 4‐chlorophenylalanine were purchased from Shanghai Aladin Co. Ltd. (Shanghai, China).

### 2.2 Human studies

Patients in this study were assigned to receive first‐line chemotherapy of pemetrexed in combination with platinum (cisplatin or carboplatin) intravenously or as maintenance therapy (two patients). Healthy controls (H) were recruited in the health management center of Nanjing Drum Tower Hospital. This study was approved by the ethics committee of Nanjing Drum Tower Hospital, affiliated hospital of medical School, Nanjing University. All patients signed written informed consent to participate in the experiment. The serum samples were prospectively collected after newly diagnosed with NSCLC and did not receive any treatment yet (ND) and after pemetrexed treatment from January 2019 to March 2019 for the discovery set and July 2021 to December 2021 for the validation set. Patients were divided into S (Effective) and R (Resistance) groups based on their sensitivity to pemetrexed treatment. The responses to chemotherapy were evaluated according to Response Evaluation Criteria in Solid Tumors (RECIST), “Effective” mean complete response or partial response, and “Resistance” mean disease progression. In the discovery set, five patients with symptoms associated with infection were excluded from the study to avoid any influence of infection on the serum metabolome. Eight patients who switched to molecular targeted therapy were also excluded. Finally, 122 serum samples were successfully selected as a discovery cohort and used for untargeted metabolomics analysis. The validation cohort containing 201 patients who met the above inclusion criteria was also recruited in Nanjing Drum Tower Hospital (nine patients with infection and 15 patients who switched to targeted molecular therapy were excluded). The validation set was used for biomarker validation and clinical biochemical analysis. All blood samples were collected fasting overnight in a blood collection tube, centrifuged at 1000 rpm for 10 min and stored at −80°C before use. The blood biochemical parameters of the validation set from each group are shown in Figure S2.

### 2.3 Sample preparation for GC–MS and LC‐Q/TOF‐MS analysis

A GC–MS‐based untargeted metabolomics method integrated with an untargeted LC‐Q/TOFMS‐based metabolomics method was used to profile metabolites in the serum of each group as previously reported.^29^, ^30^ Briefly, 100 μL of serum was extracted with 400 μL methanol containing 5 μg/mL of Myristic‐1,2‐^13^C_2_ acid as internal standard (IS) for GC–MS analysis and 15 μg/mL of 5‐^13^C‐glutamine as IS for LC‐Q/TOF‐MS analysis. After centrifugation, concentration, derivatization (for GC–MS) and re‐dissolved, the samples were detected by GC–MS, and LC‐Q/TOFMS, respectively. A pooled sample from each group was divided equally and used as quality control (QC) samples.

### 2.4 Compound identification

MS‐DIAL 3.5.2 (http://prime.psc.riken.jp) was used for peak detection, spectral deconvolution, peak alignment and compound identification. The metabolites detected by GC–MS were identified using the National Institute of Standards and Technology (NIST) library 14, Wiley 9, and an in‐house mass spectra library database as previously described.^31^, ^32^ For LC‐Q/TOF‐MS, the metabolites were identified with MS‐DIAL with the databases they provided including MassBank, GNPS, RIKEN PlaSMA authentic standards, etc. MS‐DIAL can automatically identify the metabolite peaks by the similarity calculation of retention time, precursor m/z, isotopic ratios, and MS/MS spectrum with the reference databases. A metabolite getting the “highest score” in the metabolite candidates is annotated by means of MS/MS similarity, accurate mass, isotope ratio, and retention time. If the “highest score” is less than the user‐defined identification cut‐off, “non‐MS/MS‐based identification” is performed.

### 2.5 Multivariate and univariate data analysis

Peak areas of detected compounds were normalized by the area of IS for each sample. Principal components analysis (PCA) and partial least squares discriminant analysis (PLS‐DA) was performed using the mixOmics package in the R project (version 3.6.3). The variable of importance in the project (VIP) analysis was used to identify the endogenous metabolites contributing to the classification. Furthermore, univariate analysis was conducted by one‐way ANOVA followed by a pairwise t‐test and corrected by the Benjamini‐Hochberg method to control the False Discovery Rate (FDR). The peaks with a *p*‐value less than 0.05 were considered to be differential. The differential metabolites selected by multivariate and univariate data analysis were further analyzed by fold change among ND, H, R, and S groups. The finally chosen metabolites were validated by UPLC/MSMS in the validation set.

### 2.6 Pathway analysis

Metabolomics pathway analysis and enrichment analysis of the metabolic biomarkers were carried out using the online metabolomics data analysis tool MetaboAnalyst 5.0 (www.metaboanalyst.ca)^33^ with the KEGG library and SMPDB library, respectively. The affected reactions, enzymes, modules and pathways using label propagation in a knowledge model network were generated by the “FELLA” package in the R project (version 3.6.3).

### 2.7 A targeted metabolomics method for quantitative profiling of tryptophan metabolites

A SCIEX QTRAP 5500 plus mass spectrometer (Toronto, Canada) was utilized with multiple reaction monitoring (MRM) in positive and negative ionization modes. The MS parameters were as follows: ion spray voltage 5500 V (positive mode) and − 4500 V (negative mode), source temperature (TEM) 550°C, curtain gas (CUR) 30 psi, nebulizer gas (GS1) 50 psi, heater gas (GS2) 60 psi. The MRM transitions of tryptophan, kynurenine, tryptamine, indolelactic acid, melatonin, and IS at positive mode are 206.2 → 160.0, 209.2 → 192.1, 161.1 → 144.0, 206.2 → 160.0, 233.3 → 174.2 and 200.2 → 154.0, respectively. The MRM transitions of quinolinic acid, xanthurenic acid, 3‐indolepropionic acid and IS at negative mode are 166.0 → 78.0, 204.0 → 160.0, 188.0 → 144.0 and 197.9 → 180.9, respectively. The chromatographic separation was performed using HSS T3 column (100 × 2.1 mm, 1.8 μm) from Waters (Shanghai, China). The mobile phase consisted of water containing 0.1% formic acid (mobile phase A) and acetonitrile containing 0.1% formic acid (mobile phase B). The gradient elution was programmed as follows: mobile phase B maintained at 10% (0.00 ~ 1.00 min), from 10% to 90% (1.00 ~ 9.00 min) held at 90% (9.00 ~ 12.00 min), from 90% to 10% (12.00 ~ 12.10 min) and held at 10% (12.10 ~ 15.00 min) at the flow rate of 0.3 mL/min. The temperature of the column and autosampler was controlled at 40°C and 4°C, respectively. The injection volume was set as 5 μL.

### 2.8 Analysis of tryptophan metabolites in NLCLC patients from the validation set

The working solution containing 0.1, 0.2, 0.5, 1, 2, 5, 10, 20, 50, 100 μmol/L of tryptophan and its metabolites were used to get the calibration curve. An aliquot of 100 μL serum sample from the validation set containing 201 patients was added with 400 μL methanol containing IS (4‐Chlorophenylalanine, 1 μg/mL) for the protein precipitation. After being vortexed, evaporated and redissolved with 100 μL 10% acetonitrile, 80 μL supernatant was transferred to an LC vial and 5 μL was injected for LC–MS/MS analysis.

### 2.9 The classification of different groups with a metabolite set by eight machine learning methods

The ratio was calculated between kynurenine and tryptophan (KTR), xanthurenic acid and kynurenine (XKR). Eight machine learning methods including “LogitBoost”, “lda”, “knn”, “C5.0”, “rf”, “svmRadial”, “nb”, and “nnet” were used for classification between the four groups. Models were evaluated by accuracy and the AUC. All the machine learning methods were performed and tuned using the “caret” package in the R project (version 3.6.3).

### 2.10 Random forest model

A tree‐based machine learning method, random forest, was finally selected to evaluate the efficiency of pemetrexed for treating NSCLC. The decision tree of the random forest model was established to interpret the predictions generated by the “reprtree” package in the R project (version 3.6.3).

### 2.11 Comparision of random forest models established by metabolites and clinical biochemical parameters

The blood biochemical parameters of the validation set including neutrophils, lymphocytes, fibrinogen, dimer, albumin, globulin, AG, HDLC, Apolipoprotein A, carcinoembryonic antigen, neuron‐specific enolase and NSCLC21‐1 from each group were analyzed. A random forest model was established and the AUC was calculated to evaluate the model performance and the efficiency of pemetrexed on NSCLC.

## 3 RESULTS

### 3.1 Demographics and clinical characteristics of NSCLC patients and healthy volunteers in the discovery set and validation set

One hundred and twenty‐two serum samples were selected as the discovery set, including H (*n* = 39) and patients diagnosed with NSCLC (ND group, *n* = 24, S group, *n* = 41, R group, *n* = 18). Two hundred and one serum samples, including 48 healthy volunteers and 153 NSCLC patients (ND group, *n* = 50, S group, *n* = 62, R group, *n* = 41), were selected as a validation set. All the patients received pemetrexed plus cisplatin or carboplatin as a first‐line therapy or pemetrexed as maintenance therapy. Most patients achieved disease control, including those with a confirmed partial response or stable disease. The typical CT images of the lung of patients are shown in Figure S1. The clinical characteristics of age and gender were comparable between the four groups in the discovery and validation set, which are listed in Tables 1 and 2. According to the biochemistry analysis, for all clinical biochemical parameters, compared with H and newly diagnosed NSCLC patients, no noticeable difference was observed in pemetrexed sensitive and resistant NSCLC patients other than neutrophils and lymphocytes (Table 1, Table 2, Figure S2).

**TABLE 1:** Characteristics of patients in the discovery set (*n*  = 122).

	H (*n* = 39)	ND (*n* = 24)	S (*n* = 41)	R (*n* = 18)
Age, years				
Median (range)	60 (42–75)	62 (42–83)	62 (32–75)	62 (45–79)
Sex, *n* (%)				
Male	27 (69.23%)	15 (62.50%)	28 (68.29%)	12 (66.67%)
Female	12 (30.77%)	9 (37.50%)	13 (31.71%)	6 (33.33%)
BMI (Kg/m2)				
Average	23.28 ± 2.40	22.96 ± 2.33	23.28 ± 2.16	23.04 ± 2.05
Smoking history				
Smokers	12	7	14	5
Non‐smokers	27	17	27	13
Stage				
I		2	4	1
II		1	0	0
III		3	7	2
IV		18	30	15
Neutrophils (%)	61.02 ± 5.44*	66.86 ± 9.80	60.47 ± 11.04*	70.81 ± 11.95^###^
Lymphocytes (%)	31.17 ± 4.91***	23.84 ± 8.07	27.83 ± 9.16	19.79 ± 9.59^###^

*Note* : *, *p* < 0.05; ***, *p* < 0.001, compared with ND group; ###, *p* < 0.001, compared with S group.

**TABLE 2:** Characteristics of patients in the validation set (*n*  = 201).

	H (*n* = 48)	ND (*n* = 50)	S (*n* = 62)	R (*n* = 41)
Age, years				
Median (range)	62 (42–74)	60 (39–78)	60 (39–79)	61 (44–79)
Sex, *n* (%)				
Male	31 (64.58%)	34 (68.00%)	40 (64.52%)	26 (63.41%)
Female	17 (35.42%)	16 (32.00%)	22 (35.48%)	15 (36.59%)
BMI (Kg/m2)				
Average	23.05 ± 2.58	23.00 ± 2.36	23.00 ± 2.54	23.15 ± 2.37
Smoking history				
Smokers	14	17	21	13
Non‐smokers	34	33	41	28
Stage				
I		3	6	2
II		2	1	0
III		6	9	7
IV		39	46	32
Neutrophils (%)	61.31 ± 6.31^****^	71.45 ± 11.82	57.27 ± 11.32^****^	63.49 ± 12.15^##^
Lymphocytes (%)	31.15 ± 5.42^****^	20.67 ± 11.72	29.91 ± 10.14^****^	25.8 ± 7.83^#^

*Note* : ****, *p* < 0.0001, compared with ND group; #, *p* < 0.05; ##, *p* < 0.01, compared with S group.

### 3.2 The overall analysis of metabolomics data in the discovery set

**FIGURE 1:**
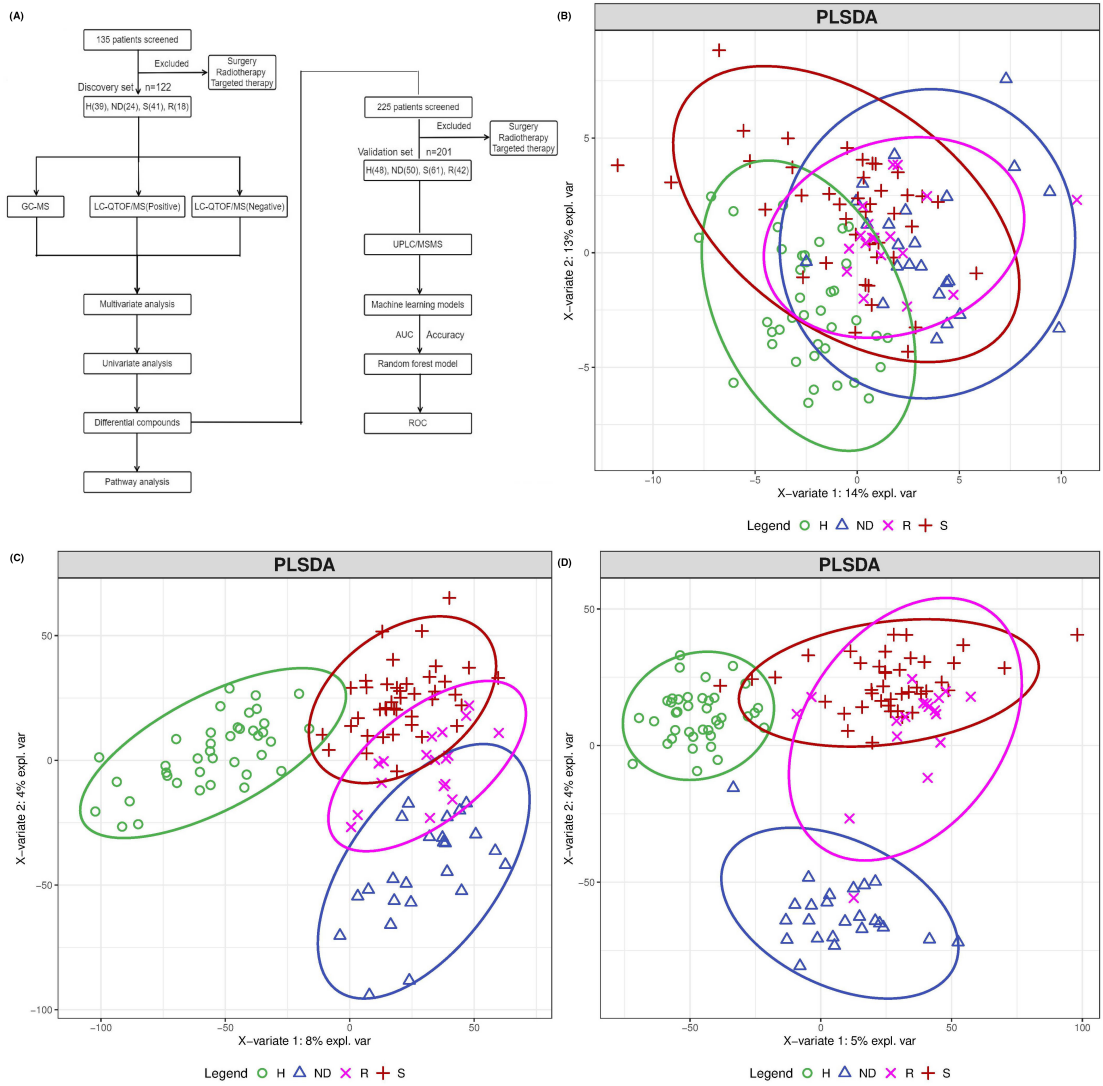
Overview of the metabolomics data from GC–MS and LC‐QTOF/MS. (A). The flowchart of the study design. Scores plots of partial least squares discriminant analysis (PLS‐DA) of H, ND, R and S for GC–MS (B) and positive (C) and negative (D) mode of LC‐QTOF/MS, respectively.

The flowchart of this study is shown in Figure 1A. Detailed information of compound identification was shown in Table S1. PCA was first used to show the overall distribution of each sample. QC samples were clustered together in the scores plot of PCA, indicating the experiment's good and robust performance, Figure S3A–C. A distinct metabolic pattern of the four groups was observed in the scores plot of PLS‐DA (Figure 1B–D), suggesting a noticeable difference in metabolites in the four groups. The detailed parameters of PCA and PLS‐DA models were shown in Table 3. The metabolic profiles of newly diagnosed NSCLC patients were well separated from those of healthy controls.

**TABLE 3:** The parameters of multivariate analysis in each model.

Mode	Type	Components	R2X(cum)	R2Y(cum)	Q2(cum)
GC	PCA(4 Groups)	3	0.417		0.305
	PLSDA(4 Groups)	4	0.433	0.405	0.198
	PLSDA(H vs. ND)	4	0.49	0.914	0.764
	PLSDA(ND vs. S)	3	0.423	0.668	0.389
	PLSDA(S vs. R)	4	0.444	0.67	0.024
POS	PCA(4 Groups)	5	0.358		0.263
	PLSDA(4 Groups)	4	0.277	0.766	0.614
	PLSDA(H vs. ND)	3	0.272	0.988	0.953
	PLSDA(ND vs. S)	4	0.251	0.985	0.828
	PLSDA(S vs. R)	4	0.253	0.979	0.708
NEG	PCA(4 Groups)	5	0.415		0.283
	PLSDA(4 Groups)	4	0.352	0.649	0.495
	PLSDA(H vs. ND)	3	0.386	0.957	0.899
	PLSDA(ND vs. S)	4	0.348	0.975	0.824
	PLSDA(S vs. R)	4	0.347	0.925	0.594

### 3.3 Differential metabolite analysis in healthy control, NSCLC, and NSCLC treated with pemetrexed (sensitive and resistant)

**FIGURE 2:**
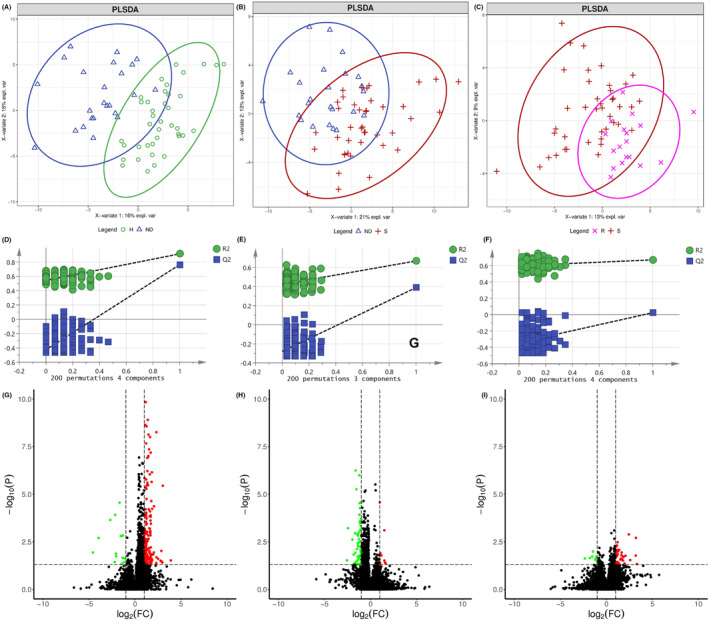
Selection of differential metabolites between H group and ND group. Scotters plots of PLSDA model of H and ND group for GC–MS (A) and positive (B) and negative (C) mode of LC‐QTOF/MS, respectively. Permutation test of PLSDA model of H and ND group for GC–MS (D) and positive (E) and negative (F) mode of LC‐QTOF/MS, respectively. Volcano plot of H and ND group for GC–MS (G) and positive (H) and negative (I) mode of LC‐QTOF/MS, respectively.

**FIGURE 3:**
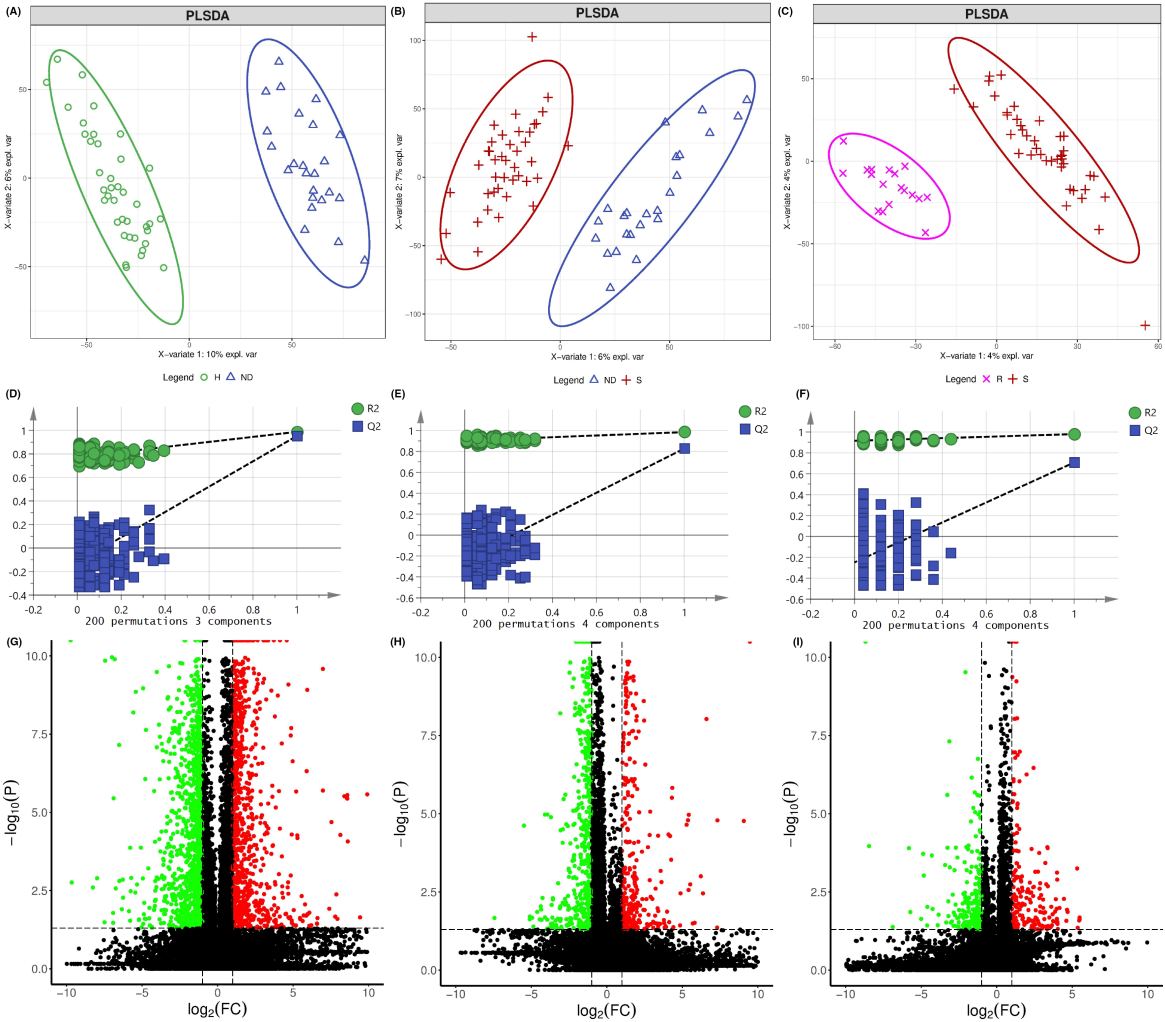
Selection of differential metabolites between ND group and S group. Scotters plots of PLSDA model of ND and S group for GC–MS (A) and positive (B) and negative (C) mode of LC‐QTOF/MS, respectively. Permutation test of PLSDA model of ND and S group for GC–MS (D) and positive (E) and negative (F) mode of LC‐QTOF/MS, respectively. Volcano plot of ND and S group for GC–MS (G) and positive (H) and negative (I) mode of LC‐QTOF/MS, respectively.

**FIGURE 4:**
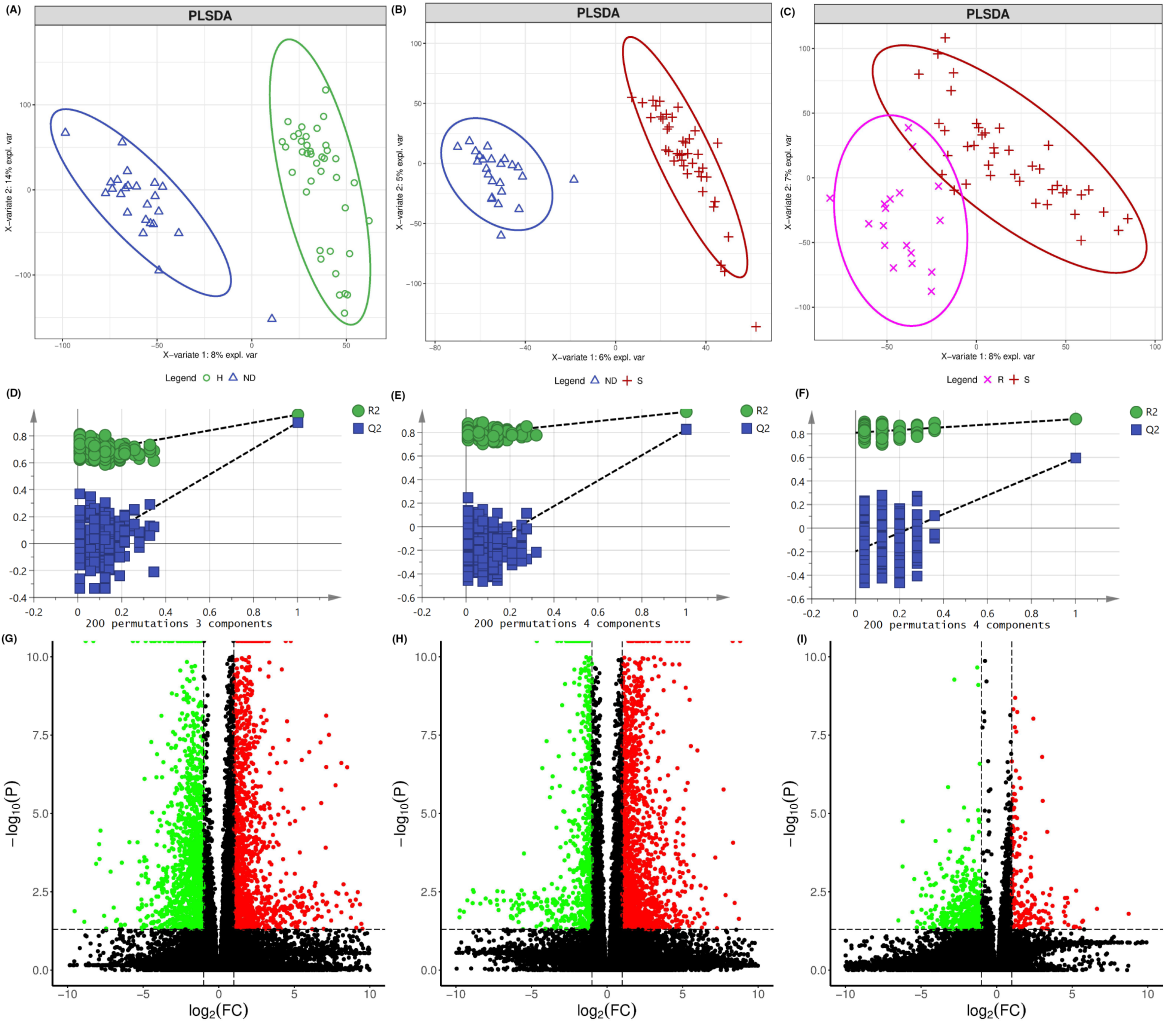
Selection of differential metabolites between R group and S group. Scotters plots of PLSDA model of R and S group for GC–MS (A) and positive (B) and negative (C) mode of LC‐QTOF/MS, respectively. Permutation test of PLSDA model of R and S group for GC–MS (D) and positive (E) and negative (F) mode of LC‐QTOF/MS, respectively. Volcano plot of R and S group for GC–MS (G) and positive (H) and negative (I) mode of LC‐QTOF/MS, respectively.

**FIGURE 5:**
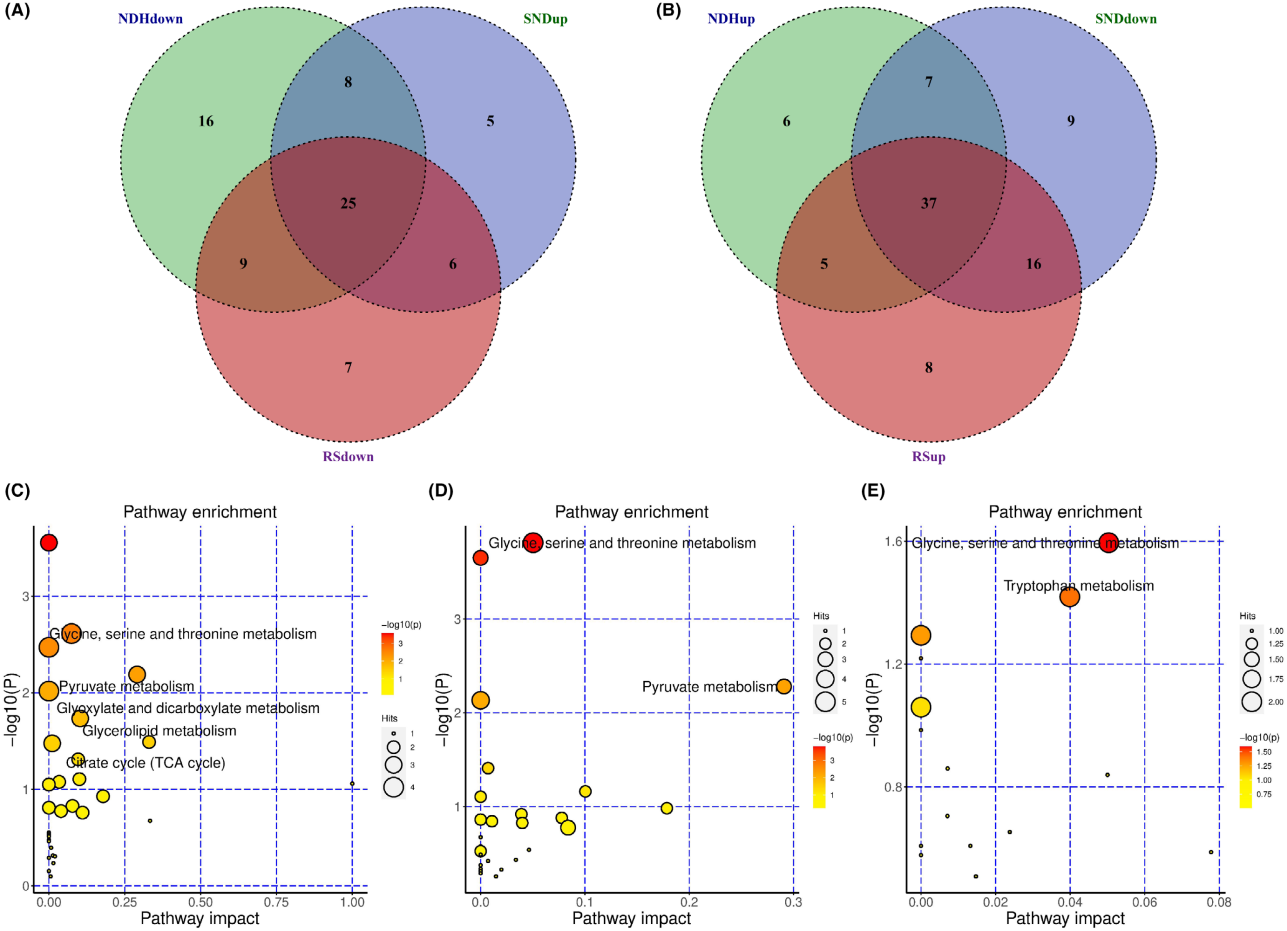
A, Venn plot of metabolites decreased in ND group compared with H group, increased in S group compared with ND group, and decreased in R group compared with S group. B, Venn plot of metabolites increased in ND group compared with H group, decreased in S group compared with ND group, and increased in R group compared with S group. The significantly changed pathways between H and ND group (C), S and ND group (D) and R and S group (E), respectively.

Differential metabolites were selected both from multivariate analysis and univariate analysis. VIP values based on the PLS‐DA model and *p*‐values of one‐way ANOVA followed by a pairwise *t*‐test and corrected by the Benjamini‐Hochberg method were used as the screening criteria. Variables with VIP values greater than 1.0 and adjusted *p*‐value (one‐way ANOVA followed by a pairwise t‐test and corrected by the Benjamini‐Hochberg method) less than 0.05 were considered differential metabolites. The detailed parameters of PCA and PLS‐DA models were shown in Table 3. The scores plots of PLS‐DA model between every two groups were shown in Figure 2A–C, Figure 3A–C and Figure 4A–C for GC–MS and positive and negative modes of LC‐QTOF/MS, respectively. The PLS‐DA models were validated by permutation test and shown in Figure 2D–F, Figure 3D–F, and Figure 4D–F, respectively. The volcano plot showed the increased and decreased metabolites in the ND group compared with the H group (Figure 2G–I), S group compared with ND group (Figure 3G–I), and R group compared with the S group (Figure 4G–I) in GC–MS and LC‐TOF/MS (ESI positive mode and negative mode), respectively. Twenty‐five metabolites with a decreasing trend in the ND group compared with the H group, an increasing trend in the S group compared with the ND group, a decreasing trend in the R group compared with S group (Figure 5A), or 37 metabolites with opposite directions were shown in the Venn plot (Figure 5B).

### 3.4 Pathway analysis of differential metabolites

The selected most important metabolites in serum among each group were further analyzed by the online tool MetaboAnalyst 5.0 (https://www.metaboanalyst.ca) to reveal the key pathways changed after pemetrexed treatment. The metabolites were classified by their structure into different classes. Amino acids, saturated fatty acids, unsaturated fatty acids, TCA acids, tetracarboxylic acids, and dicarboxylic acids were the most enriched metabolite classes (Figure S4A). The selected metabolites were mapped to KEGG metabolic pathways for overrepresentation and pathway analysis (Figure 4). Glycine, serine, and threonine metabolism, pyruvate metabolism, glyoxylate and dicarboxylate metabolism, glycerolipid metabolism and TCA cycle were the most significantly changed pathways in ND group compared with H group (Figure 5C). Glycine, serine, and threonine metabolism and pyruvate metabolism were the most changed considerably pathways in S group compared with ND group (Figure 5D). Glycine, serine and threonine metabolism and tryptophan metabolism were the most altered pathways among S and R group (Figure 5E). Further, a knowledge model network generated by the “FELLA” package in the R project showed that the PI3K‐Akt signaling pathway, central carbon metabolism in cancer and mineral absorption were the most affected pathways in ND group compared with H group (Figure S4A). Central carbon metabolism in cancer and mineral absorption were the most affected pathways in S group compared with ND group (Figure S4B). Pantothenate and CoA biosynthesis, epithelial cell signaling and mineral absorption were the most affected pathways in R group compared with S group (Figure S4C).

### 3.5 Biomarkers validation in the validation set for the evaluation of pemetrexed response

**FIGURE 6:**
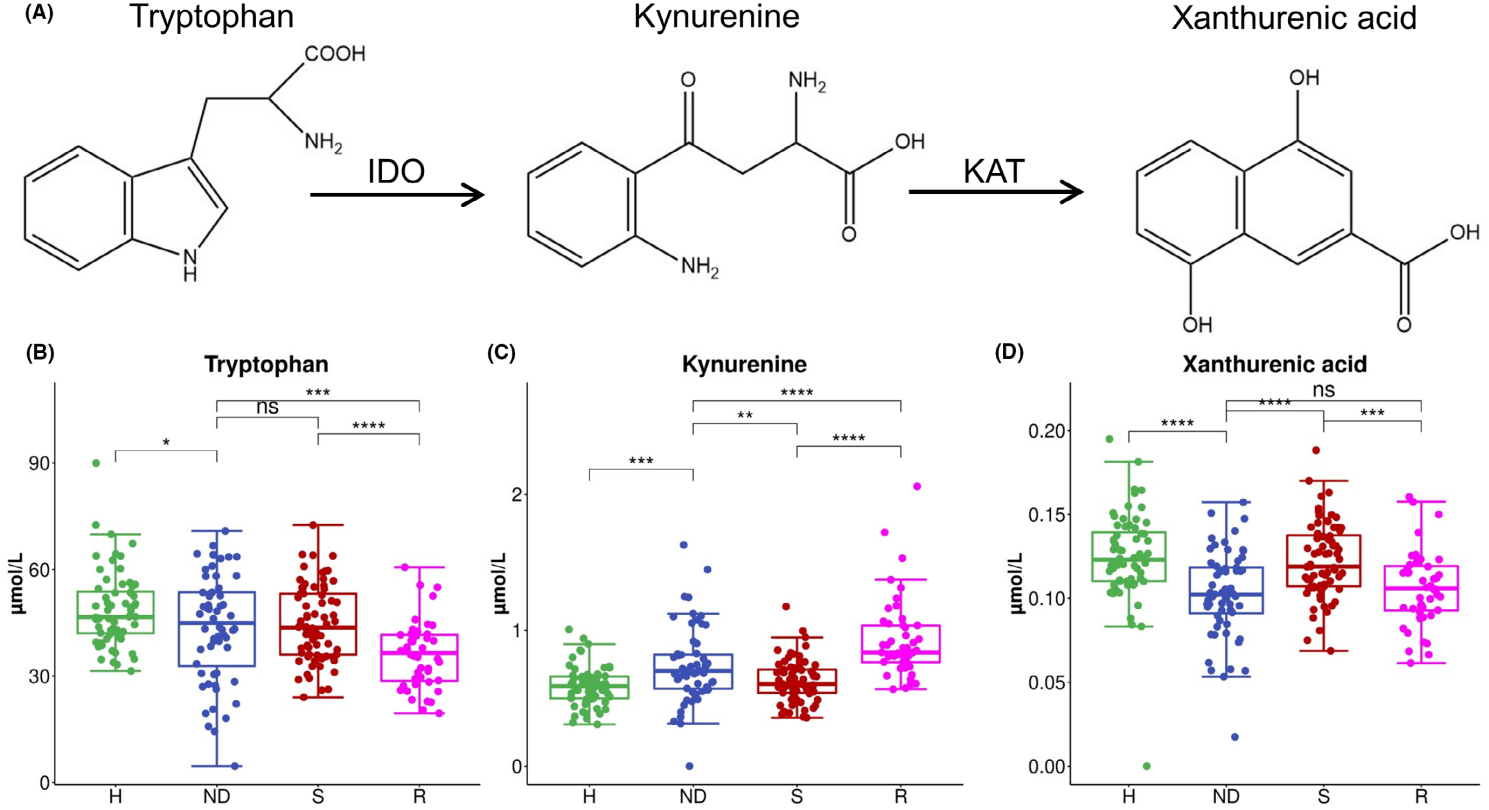
The metabolic pathway of tryptophan metabolism (A) and Box plot of metabolites selected as the potential biomarkers for evaluating pemetrexed efficiency (B, trypthan, C, kynurenine, D, xanthurenic acid).

The citrate cycle, pyruvate metabolism, and lipids may be influenced by food intake and exercise, thus we selected tryptophan metabolism to validate pemetrexed response in NSCLC patients. We established a targeted metabolomics method referenced from previous literature using UPLC–MS/MS.^34^ We measured the concentrations of eight metabolites (tryptophan, kynurenine, xanthurenic acid, tryptamine, indolelactic acid, melatonin, quinolinic acid, and 3‐indolepropionic acid) in tryptophan metabolism in the validation set. The chromatograms of tryptophan metabolites in positive and negative modes were shown in Figures [Link], [Link], respectively. The concentration of kynurenine increased in the ND group compared with the H group, and decreased after effective pemetrexed treatment but did not change in the ineffective pemetrexed treatment group. Tryptophan and xanthurenic acid had an opposite trend (Figure 6).

### 3.6 Machine learning models selection for evaluating pemetrexed efficiency

**FIGURE 7:**
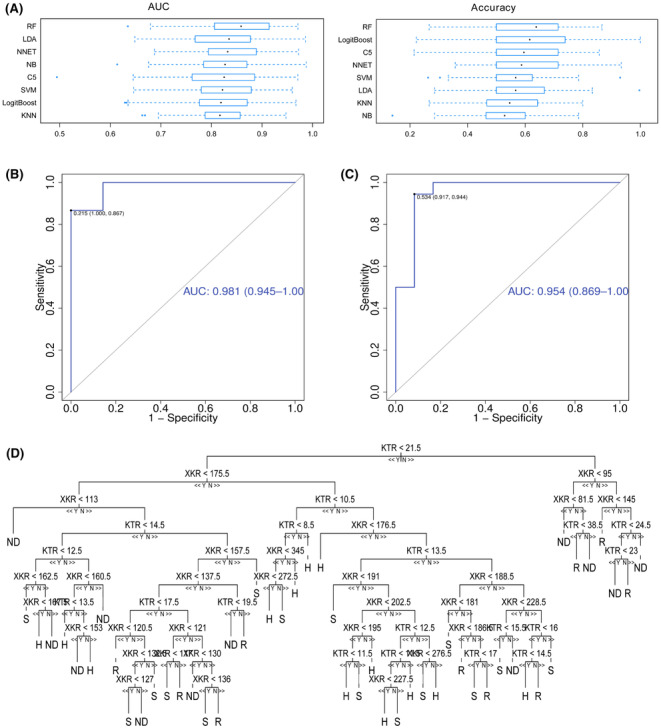
Comparison of eight machine learning models used for evaluation of pemetrexed efficiency and decision tree of random forest model. (A), Accuracy and AUC of machine learning models used to assess pemetrexed efficiency. (B) the AUC of ROC for diagnosing NSCLC, (C) the AUC of ROC for evaluating the efficiency of pemetrexed, (D) The decision trees represent the random forests machine learning algorithm.

To accurately evaluate the efficiency of pemetrexed for treating NSCLC, eight machine learning methods with three metabolites were performed and compared. We performed 10‐fold cross‐validation on the training set, and average accuracy, and AUC were calculated to evaluate the model performance. The random forest method had the maximum average accuracy and AUC (Figure 7A). Thus we selected the random forest method to establish the final evaluation model. The random forest model randomly created decision trees consisting of KTR and XKR were used to generate the final output. The AUC of H versus ND and R versus S is 0.981 and 0.954, respectively (Figure 7B, C). The figure shows the decision tree generated by three metabolites from the random forest model (Figure 7D). The efficiency of pemetrexed can be evaluated by the relative abundance of the ratio between the three metabolites step by step.

### 3.7 Random forest model established by metabolites outperformed clinical biochemical parameters

**FIGURE 8:**
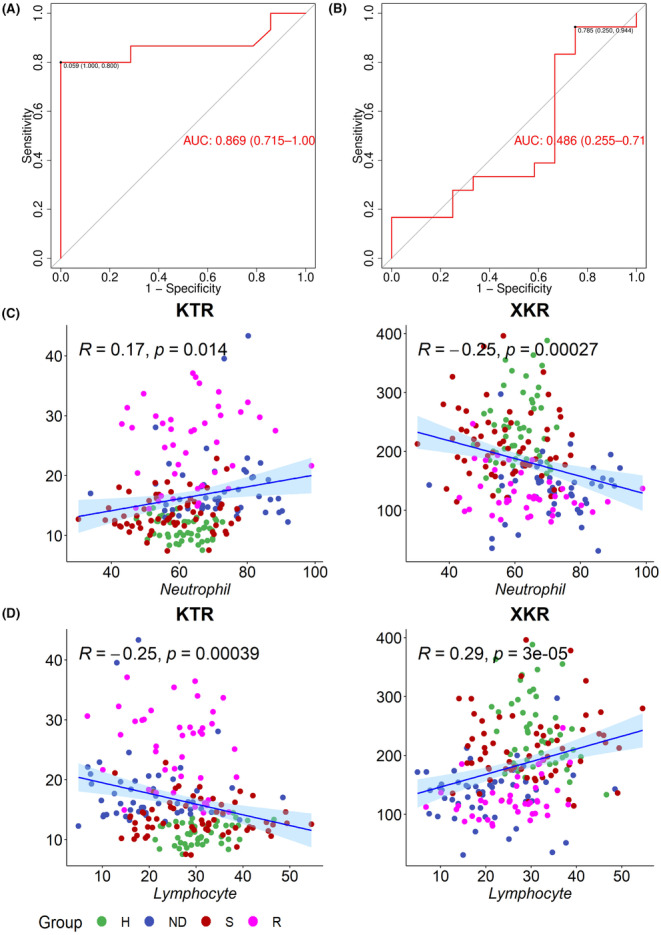
Random forest model established by using neutrophils and lymphocytes. **(**A) the AUC of the random forest model established by using neutrophils and lymphocytes to distinguish the ND group and the H group. (B) the AUC of the random forest model established by using neutrophils and lymphocytes to distinguish the S group and the R group. (C) the correlation of neutrophils with KTR and XKR. (D) the correlation of lymphocytes with KTR and XKR.

For all clinical biochemical parameters, the AUC of the random forest model established with neutrophils and lymphocytes is 0.869 and 0.486 for distinguishing the ND group versus the H group and the S group versus the R group, respectively (Figure 8A, B). An obvious but moderate correlation was observed between neutrophils and lymphocytes with KTR and XKR, respectively (Figure 8C, D). The OOB error rates between H and ND group, ND and S group and S and R group are 15.62%, 16.07%, and 14.56%, respectively. The random forest model established by KTR and XKR is far better than that established by neutrophils and lymphocytes.

## 4 DISCUSSION

Pemetrexed is a multitargeted antifolate drug approved as a part of first‐line NSCLC therapy. It improves the overall survival of patients with advanced NSCLC.^35^ Despite its efficiency, resistance caused by multiple reasons is a big problem.^36^ To explore the panoramic view of metabolic change in NSCLC patients and establish a classification model for evaluating pemetrexed efficiency, we recruited NSCLC patients sensitive or resistant to pemetrexed treatment. The metabolic ability was disturbed in patients diagnosed with NSCLC and showed significant differences in patients sensitive or resistant to pemetrexed.

Metabolic alteration is proposed to occur in patients diagnosed with NSCLC. The previous studies showed that amino acids, fatty acids, purines, and lipids are disturbed in NSCLC.^7^, ^28^ Our previous study also observed a significant change in metabolic pathways, including arginine biosynthesis, alanine, aspartate and glutamate metabolism, valine, leucine and isoleucine biosynthesis, and TCA cycle in newly diagnosed NSCLC.^37^ Another study found that seven metabolites, including hypotaurine, uridine, dodecanoylcarnitine, choline, dimethylglycine, niacinamide, and L‐palmitoylcarnitine were associated with chemo response of pemetrexed in NSCLC.^38^ In this study, we found that the most changed pathways in newly diagnosed patients with NSCLC were tryptophan metabolism, glycine, serine and threonine metabolism, pyruvate metabolism, and the TCA cycle. Increased serum kynurenine/tryptophan ratio correlated with disease progression in NSCLC^39^ and have prognostic and predictive values in patients with solid tumors treated with immunotherapy.^40^ Here we also observed an increase in metabolism from tryptophan to tryptamine, which may be considered a biomarker for the efficiency of pemetrexed.^41^ Tumor cells prefer anaerobic metabolism even under sufficient oxygen conditions, the Warburg Effect,^42^ an important reason for tumor proliferation and migration. The PI3K‐Akt signaling pathway is the downstream pathway influenced by pemetrexed treatment in NSCLC. PI3K/AKT pathway regulates multiple cellular functions, including cell growth, differentiation, proliferation, survival, motility, and invasion.^43^, ^44^ It has been reported that the blockade of PI3K might maximize the IFN‐γ‐mediated antitumor effect.^45^ Several PI3K/Akt inhibitors are currently under development for treating NSCLC. Pemetrexed may function on the PI3K/AKT pathway and may be a sensitive marker for its efficiency.

To further validate the biomarkers selected for diagnosis of NSCLC and evaluation of pemetrexed's efficacy, we developed a UPLC–MS/MS based targeted metabolomics method to measure concentrations of tryptophan metabolites in the validation set. Consistent with the result of untargeted metabolomics, an increase of kynurenine and a decrease of tryptophan and xanthurenic acid were observed in NSCLC patients and patients resistant to pemetrexed. It has been reported that decreased tryptophan and increased kynurenine/tryptophan ratio were associated with lung cancer risk.^46^ However, to our knowledge, the influence of pemetrexed on tryptophan metabolism and its relationship with pemetrexed efficiency were unknown. Indolamine 2,3‐dioxygenase 1 (IDO1) is the rate‐limiting enzyme of the tryptophan‐kynurenine pathway. Inhibitors of IDO1 are widely applied in several clinical trials to overcome cancer immune escape and improve therapeutic potential.^47^ Here we found that increased kynurenine/tryptophan ration and decreased xanthurenic acid/kynurenine ratio are closely related to pemetrexed's efficiency. The tryptophan metabolism pathway may be a sensitive biomarker to evaluate pemetrexed's efficiency accurately for treating NSCLC, and targeting tryptophan metabolism may be a promising way to improve pemetrexed's efficiency.

Artificial intelligence and machine learning have been widely applied in medical and pharmaceutical science.^48^, ^49^ Previously we used a random forest regression model to predict the liver weight recovery after 2/3 hepatectomy.^50^ Here we aimed to establish a classification model between metabolites in the serum of NSCLC patients who were sensitive and resistant to pemetrexed, and further select the potential biomarkers for evaluating pemetrexed efficiency. We compared eight machine learning algorithms in “caret” package of R project for classification. The comparison of average accuracy and AUC of the methods used showed RF is the most accurate method. NNET is much more time‐consuming and hard to interpret, and the decisions cannot be explained. Thus we select RF as the final method used to analyze pemetrexed efficiency. A metabolite set containing tryptophan, kynurenine, and xanthurenic acid was chosen as the potential biomarkers for evaluating pemetrexed efficiency in NSCLC. The model was easy to be performed and had the potential to be transformed into clinical application.

There remain some shortages of this study. First, the sample size of patients recruited in this study is limited, and a largepatient scale should be recruited to validate the mathematical model and accurately evaluate pemetrexed's efficiency. Second, this is a single‐center study, and more centers should be included to verify the effectiveness of the metabolites and machine learning method. Third, a mechanism study should be performed to reveal the reason for metabolic change for pemetrexed sensitivity and resistance. Last, an approach targeting metabolism to improve pemetrexed efficiency should be considered for benefit in treating NSCLC.

## 5 CONCLUSIONS

In conclusion, by using a combination of high‐throughput GC/MS‐based and LC‐QTOF/MS‐based untargeted metabolomics technologies, and LC‐MS/MS‐based targeted metabolomics technologies and machine learning algorithms, we establish eight mathematical models to evaluate pemetrexed efficiency for treating NSCLC and further compare their performance. We finally chose an explainable RF method over other methods and a set of three metabolites containing tryptophan, kynurenine and xanthurenic acid outperformed the clinical biochemical parameters neutrophils and lymphocytes to evaluate pemetrexed efficiency. In the future, we plan to validate the model in more centers and develop a method targeting metabolism to improve the efficiency of pemetrexed for treating NSCLC.

## AUTHOR CONTRIBUTIONS

**Runbin Sun:** Conceptualization (equal); data curation (equal); software (equal); visualization (equal); writing – original draft (lead). **Fei Fei:** Methodology (equal). **Min Wang:** Methodology (equal). **Junyi Jiang:** Methodology (equal); resources (equal). **Guangyu Yang:** Methodology (equal); resources (equal). **Na Yang:** Methodology (equal). **Dandan Jin:** Methodology (equal). **Zhi Xu:** Methodology (equal). **Bei Cao:** Project administration (equal); writing – review and editing (equal). **Juan Li:** Funding acquisition (equal); project administration (equal); writing – review and editing (equal).

## FUNDING INFORMATION

This research was funded by the Medical Science and Technology Development Foundation, Nanjing Department of Health (YKK21081, YKK21076), the National Natural Science Foundation of China (31371399, 82274004, 82204501), Natural Science Foundation of Nanjing University of Chinese Medicine ( XZR2020055, XZR2021089, XZR2021090).

## CONFLICT OF INTEREST STATEMENT

The authors declare no conflict of interest.

## Supporting information


**Supplementary Figure 1.** The typical CT image of the lung from NSCLC patients sensitive or resistant to pemetrexed treatment.

**Supplementary Figure 2.** Blood biochemical parameters in all groups.

**Supplementary Figure 3.** Scores plots of principal component analysis (PCA) of H, ND, R and S group for GC–MS (A) and positive (B) and negative (C) mode of LC‐QTOF/MS, respectively. D, the box plot of differential metabolites.

**Supplementary Figure 4.** A knowledge model network generated by the “FELLA” package between H group and ND group (A), ND group and S group (B) and S group and R group (C), respectively.

**Supplementary Figure 5.** The typical chromatography of tryptophan metabolites in positive mode.

**Supplementary Figure 6.** The typical chromatography of tryptophan metabolites in negative mode.

**Supplementary Table 1.**The detailed information of compound identification and pathway analysis.

## Data Availability

The data used to support the findings of this study are available either online or from the corresponding author upon request.
