# Supplementary figures and images for: Integration of metabolomics and machine learning revealed tryptophan metabolites are sensitive biomarkers of pemetrexed efficacy in non‐small cell lung cancer
Source: Cancer Med. 2023 Aug 21;12(18):19245–59. doi: 10.1002/cam4.6446 (PMC10557891; doi:10.1002/cam4.6446)

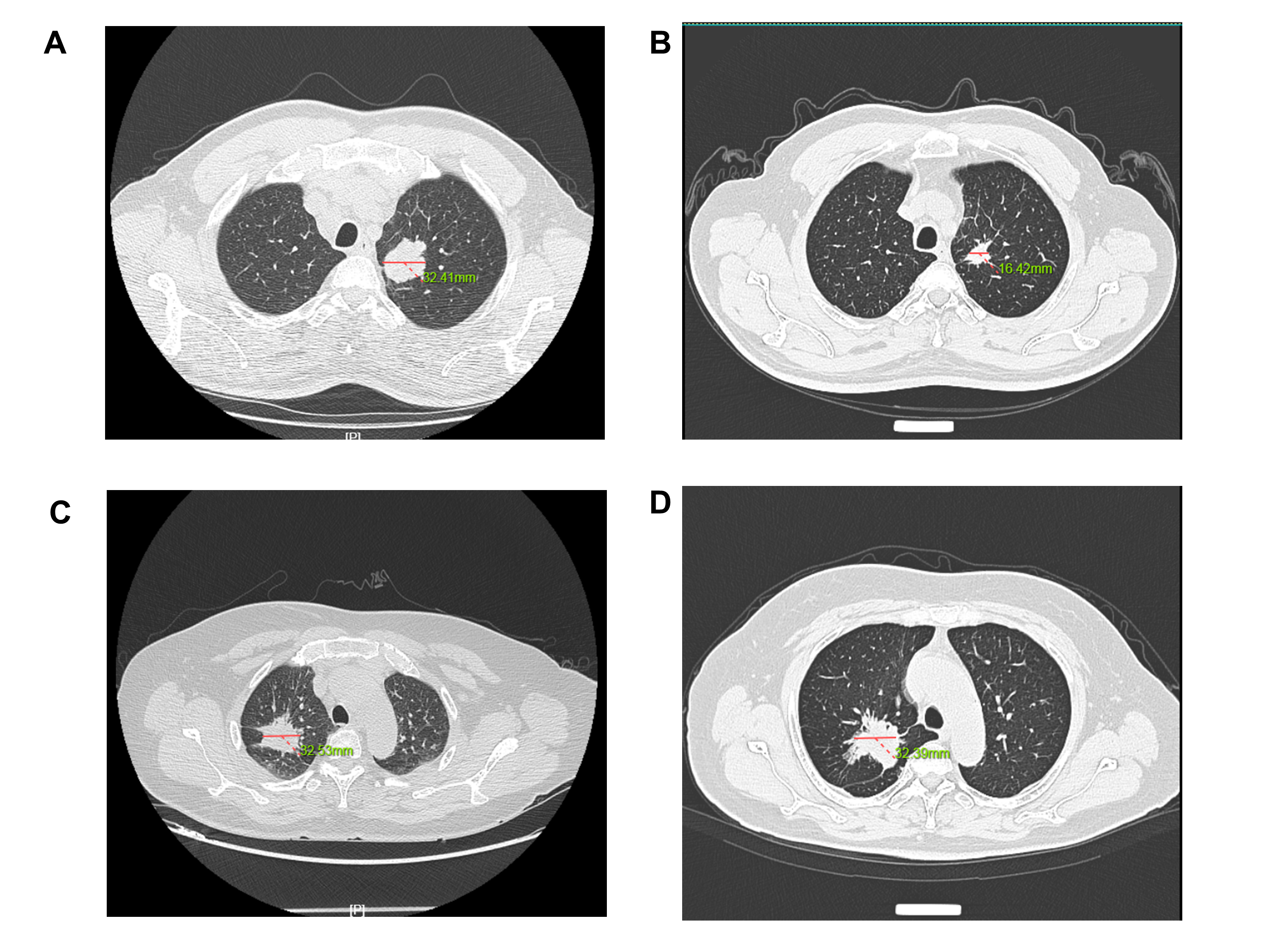

Supplement: Supplementary file 1 — Supplementary Figure 1. The typical CT image of the lung from NSCLC patients sensitive or resistant to pemetrexed treatment. [file CAM4-12-19245-s001.tif]

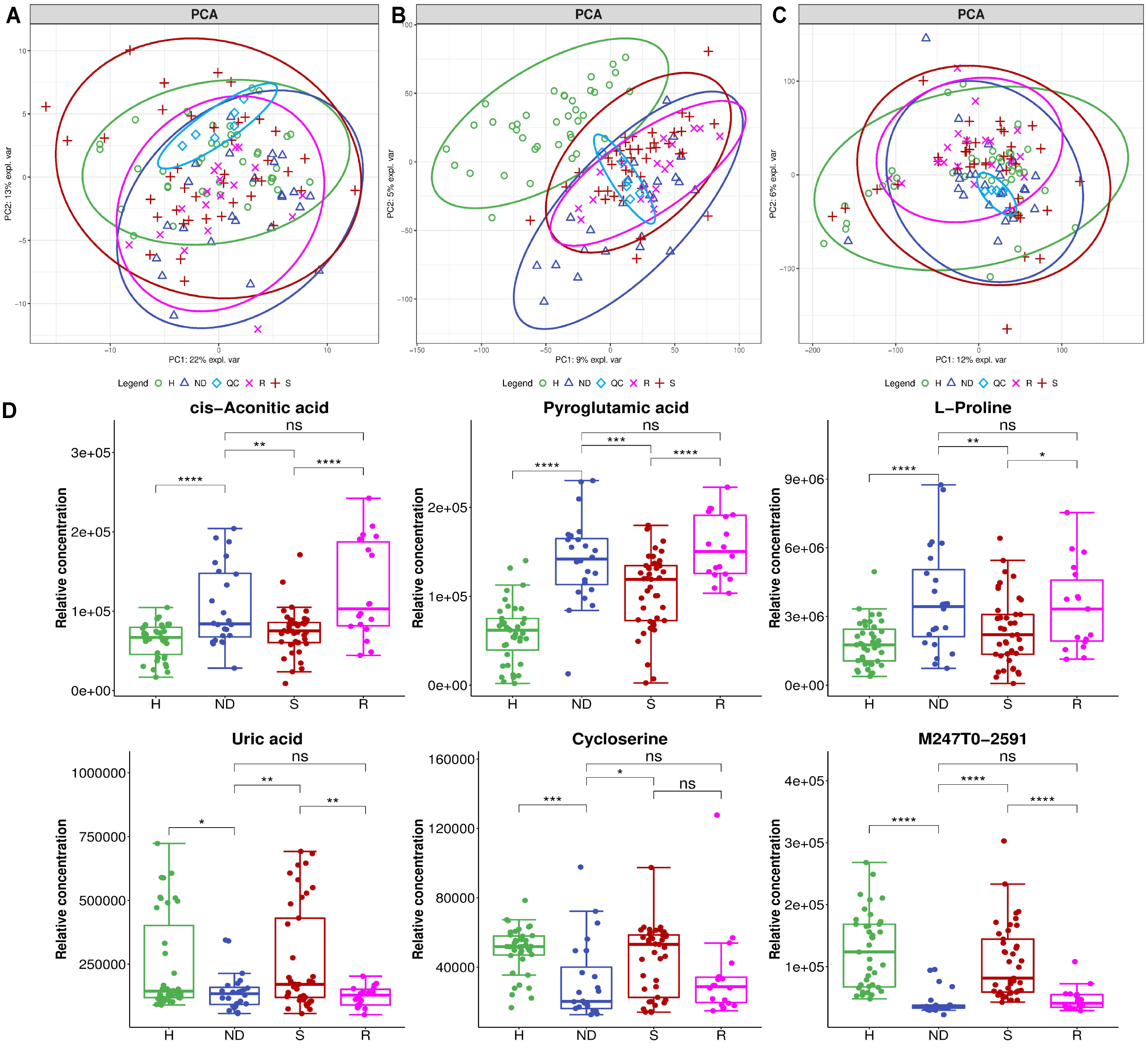

Supplement: Supplementary file 3 — Supplementary Figure 3. Scores plots of principal component analysis (PCA) of H, ND, R and S group for GC–MS (A) and positive (B) and negative (C) mode of LC‐QTOF/MS, respectively. D, the box plot of differential metabolites. [file CAM4-12-19245-s006.tif]

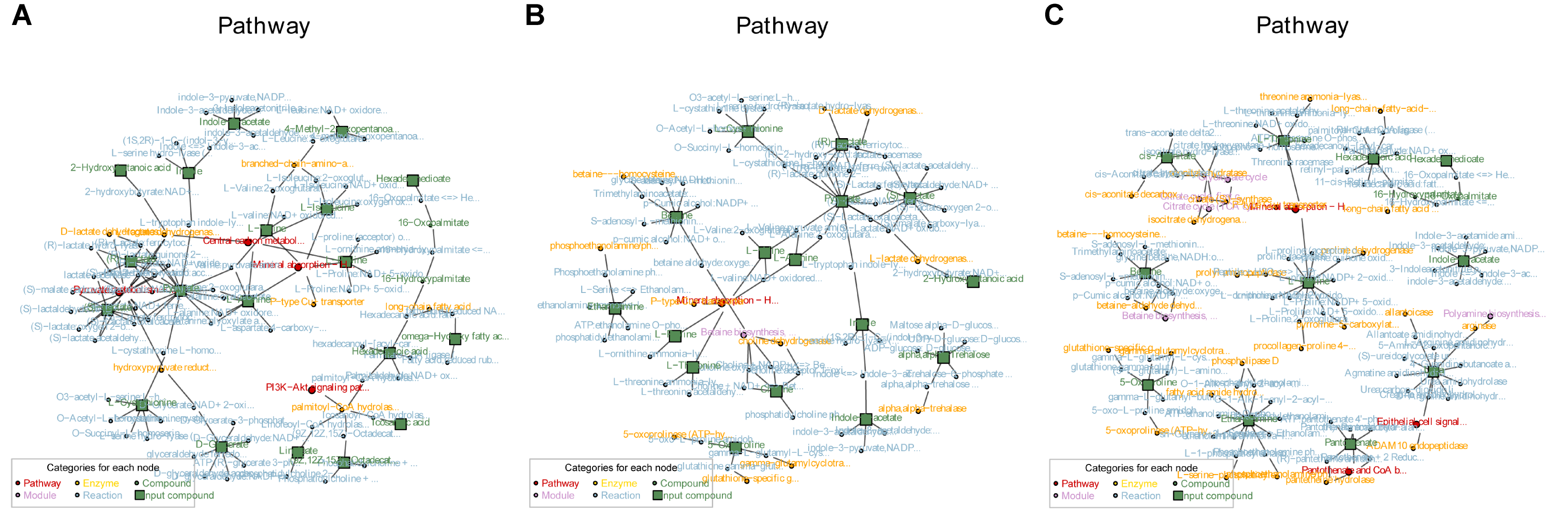

Supplement: Supplementary file 4 — Supplementary Figure 4. A knowledge model network generated by the “FELLA” package between H group and ND group (A), ND group and S group (B) and S group and R group (C), respectively. [file CAM4-12-19245-s007.tif]

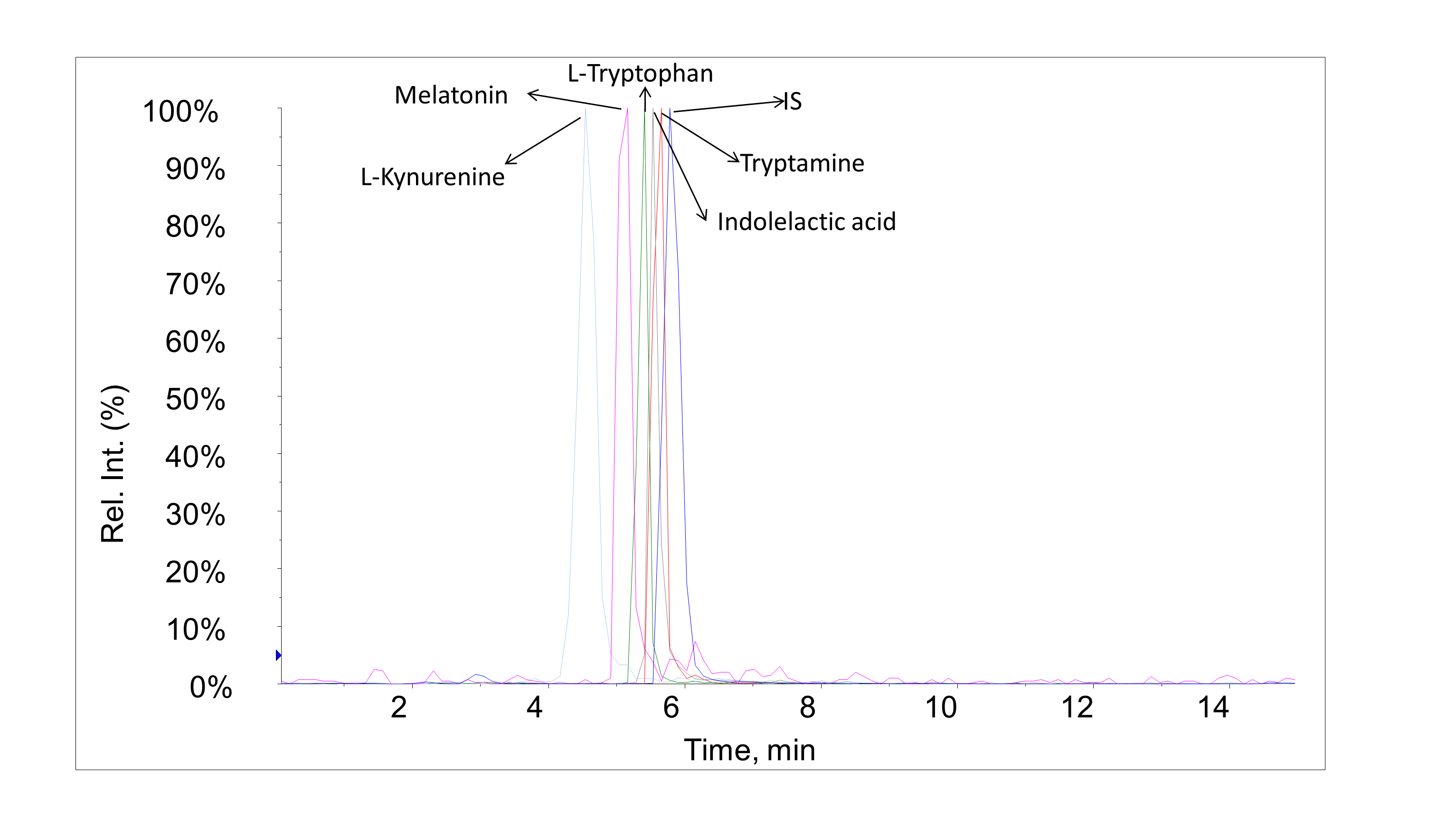

Supplement: Supplementary file 5 — Supplementary Figure 5. The typical chromatography of tryptophan metabolites in positive mode. [file CAM4-12-19245-s003.tif]

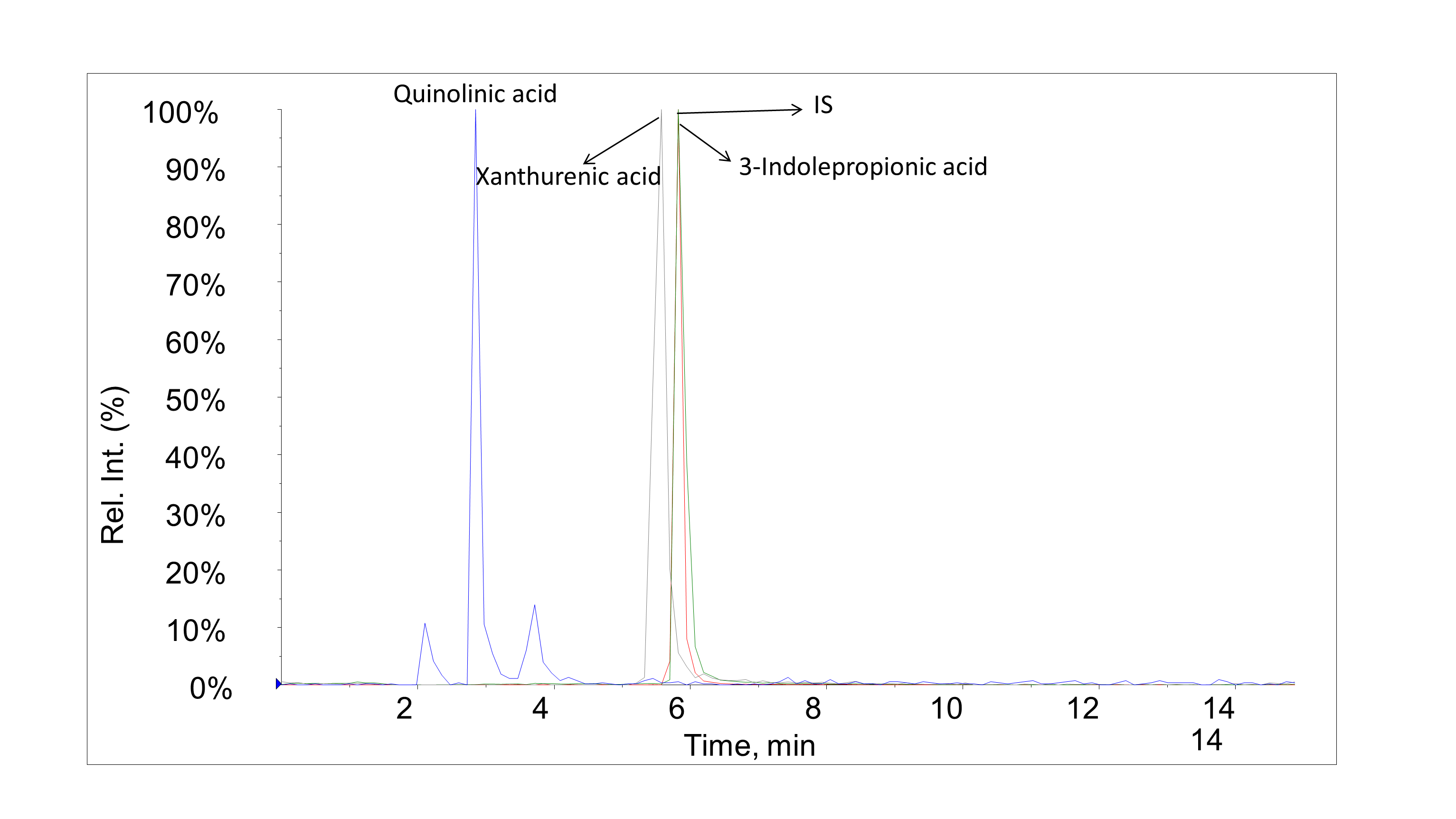

Supplement: Supplementary file 6 — Supplementary Figure 6. The typical chromatography of tryptophan metabolites in negative mode. [file CAM4-12-19245-s004.tif]
